# Pathological Cyclic Strain-Induced Apoptosis in Human Periodontal Ligament Cells through the RhoGDIα/Caspase-3/PARP Pathway

**DOI:** 10.1371/journal.pone.0075973

**Published:** 2013-10-10

**Authors:** Li Wang, Jinsong Pan, Tingle Wang, Meng Song, Wantao Chen

**Affiliations:** 1 Department of Stomatology, First People’s Hospital, Shanghai Jiao Tong University School of Medicine, Shanghai, China; 2 Department of Oral and Maxillofacial Surgery, Ninth People’s Hospital, Shanghai Jiao Tong University School of Medicine, Shanghai Key Laboratory of Stomatology and Shanghai Research Institute of Stomatology, Shanghai, China; 3 Department of Stomatology, Central Hospital of Minhang District, Shanghai, China; Boston University Goldman School of Dental Medicine, United States of America

## Abstract

**Aim:**

Human periodontal ligament (PDL) cells incur changes in morphology and express proteins in response to cyclic strain. However, it is not clear whether cyclic strain, especially excessive cyclic strain, induces PDL cell apoptosis and if so, what mechanism(s) are responsible. The aim of the present study was to elucidate the molecular mechanisms by which pathological levels of cyclic strain induce human PDL cell apoptosis.

**Materials and Methods:**

Human PDL cells were obtained from healthy premolar tissue. After three to five passages in culture, the cells were subjected to 20% cyclic strain at a frequency of 0.1 Hz for 6 or 24 h using an FX-5000T system. Morphological changes of the cells were assessed by inverted phase-contrast microscopy, and apoptosis was detected by fluorescein isothiocyanate (FITC)-conjugated annexin V and propidium iodide staining followed by flow cytometry. Protein expression was evaluated by Western blot analysis.

**Results:**

The number of apoptotic human PDL cells increased in a time-dependent manner in response to pathological cyclic strain. The stretched cells were oriented parallel to each another with their long axes perpendicular to the strain force vector. Cleaved caspase-3 and poly-ADP-ribose polymerase (PARP) protein levels increased in response to pathological cyclic strain over time, while Rho GDP dissociation inhibitor alpha (RhoGDIα) decreased. Furthermore, knock-down of RhoGDIα by targeted siRNA transfection increased stretch-induced apoptosis and upregulated cleaved caspase-3 and PARP protein levels. Inhibition of caspase-3 prevented stretch-induced apoptosis, but did not change RhoGDIα protein levels.

**Conclusion:**

The overall results suggest that pathological-level cyclic strain not only influenced morphology but also induced apoptosis in human PDL cells through the RhoGDIα/caspase-3/PARP pathway. Our findings provide novel insight into the mechanism of apoptosis induced by pathological cyclic strain in human PDL cells.

## Introduction

During occlusal load or orthodontic tooth movement, the cells in the periodontal ligament (PDL) are directly subjected to mechanical stress. The response to mechanical stress is an essential biological reaction [1], [2], [3], [4]. Prediction of tooth mobility under functional loads is a classic issue in dental biomechanics and is especially important in the development of new solutions for dental restoration, prosthodontics, and orthodontic treatment. The understanding of tooth mobility requires mechanical characterisation of the PDL. The PDL is a complex soft tissue that connects teeth to the surrounding bone, and a common assumption is that it acts as the major element in tooth mobility and stress distribution to supporting tissues [5], [6].

Apoptosis induced by cyclic strain is an important determinant of connective tissue destruction in periodontal disease [7]. The application of light orthodontic force causes direct resorption of alveolar bone and tooth mobility, while the application of excessive orthodontic force results in excessive cyclic strain, which induces local ischaemia, tissue hyalinisation, and cell death in the PDL [8]. Cells undergo death by two major mechanisms: necrosis, in which primary damage to the metabolic or membrane integrity of the cell occurs, and apoptosis, which is an internal suicide program contained in all cells [9]. Programmed cell death (apoptosis) [10], [11] plays a key role in the regulation of tissue turnover in long-lived mammals that must integrate multiple physiological as well as pathological death signals.

Many apoptotic signalling pathways have been identified, including the Fas/FasL pathway, the caspase family pathway, the cytochrome C signalling pathway, and the mitochondrial pathway [12], [13], [14], [15]. Of these apoptotic signalling pathways, the caspase family pathway is considered to be of great importance because many signalling pathways ultimately activate caspase cascades. Caspases are cysteine protease family members [16] and play an essential role in apoptosis [17], [18]. Activated caspases can initiate protein degradation and cell apoptosis irreversibly by cleaving substrate proteins such as poly-ADP-ribose polymerase (PARP).

Rho family proteins participate in the regulation of polarity, proliferation, adhesion, spreading, migration, cytoskeleton organisation, and apoptosis of cells. Rho GDP dissociation inhibitor alpha (RhoGDIα) is frequently overexpressed in human tumours and chemoresistant cancer cell lines, raising the possibility that RhoGDIα is an anti-apoptotic molecule in cancer cells [19]. In normal cells, a previous study showed that RhoGDIα plays a critical role in low shear stress-induced apoptosis of vascular smooth muscle cells [20]. Hence, it was hypothesised that RhoGDIα may participate in apoptosis of other normal cells such as human PDL cells.

Until now, no *in vitro* experiments have clearly investigated the mechanism of apoptosis of human PDL cells under pathological conditions of cyclic strain. In this study, we evaluated the roles of RhoGDIα, caspase-3, and PARP proteins in cyclic stretch-induced apoptosis of human PDL cells. First, we investigated the relationship among cyclic stretch, cell morphology, and apoptosis by subjecting human PDL cells to pathological levels of cyclic stretching force (20% cyclic strain) [21], [22] for 6 and 24 h. Immediately after the application of strain, we evaluated the extent of apoptosis to determine how time under strain affected human PDL cells. We used inverted phase-contrast microscopy to observe the morphology of apoptotic cells and flow cytometry to count the number of apoptotic cells in each treatment group. Second, we investigated the roles of RhoGDIα, caspase-3, and PARP proteins in human PDL cell apoptosis using Western blot analysis. We provide novel insight into the mechanism of apoptosis induced by pathological cyclic strain in human PDL cells through the activation of caspase-3 via the RhoGDIα signalling cascade.

## Materials and Methods

### Antibodies and Reagents

The antibodies used for the Western blot and immunocytochemistry included monoclonal anti-cytokeratin (D1E4) (1∶500), monoclonal anti-vimentin (D21H3) (1∶500), monoclonal anti- caspase-3 (8G10) (1∶500), and monoclonal anti-PARP (46D11) (Cell Signaling, Beverly, MA, USA). Polyclonal anti-RhoGDIα antibody (1∶500) was from Sigma (St. Louis, MO, USA).

### Ethics Statement

The study protocol was approved by the Ethics Committee of Shanghai Jiao Tong University (China), and written informed consent was obtained from each donor’s parents in accordance with the Declaration of Helsinki (n = 3, including one 13-year-old females and two 14-year-old males). The results of the individual cell lines has been provided as Figures S1, S2, S3, S4, S5, S6.

### Cell Culture and Treatment

Human PDL cells were obtained from healthy premolar tissues following orthodontic extraction, as described previously [23]. PDL tissue was removed from the middle third of the root using a sterile scalpel and rinsed five times in Dulbecco’s Modified Eagle’s Medium (DMEM; Gibco, Life Technologies, Grand Island, NY, USA). PDL tissues from one donor were attached to a flank in one culture bottle. PDL tissues were digested with 0.25% trypsin for 5 min and collected by centrifugation at 800 rpm for 6 min. The residue was incubated with 0.2% collagenase I for 50 min at 37°C in a shaker. After centrifugation at 800 rpm for 3 min, the residue was added to DMEM containing 20% (v/v) foetal bovine serum (FBS; Hyclone, Logan, UT, USA) and antibiotics (100 U ml^−1^ penicillin plus 100 mg ml^−1^ streptomycin) by pipetting repeatly. The cells grown out were cultured in growth medium at 37°C in a humidified atmosphere of 5% CO_2_ and passaged in 10% FBS/DMEM supplemented with antibiotics (100 U ml^−1^ penicillin plus 100 mg ml^−1^ streptomycin). Cells of passages 4–8 were used in all experiments. Passage 4 cells were stained with anti-vimentin and anti-cytokeratin antibodies for characterisation.

For cyclic strain experiments, human PDL cells were seeded onto collagen I-coated six-well Bioflex plates (Flexcell International, Hillsborough, NC, USA) at a density of 3×10^5^ cells/well. Cells at 95% confluence were serum-starved in DMEM for 24 h and then subjected to cyclic strain using a Flexcell Tension Plus system (FX-5000T; Flexcell International) with a 20% elongation magnitude at the frequency of 0.1 Hz for 6 or 24 h, respectively. Cells cultured on the same kind of plates without stretch loading, i.e., a static group, were used as time-matched static control cells.

### Analysis of Morphological Changes

The morphologies of the PDL cells before and after cyclic strain loading and the PDL cells in the static group were observed under an inverted phase-contrast microscope (Leica DMIRB; Leica Microsystems, Bensheim, Germany).

### Measurement of Apoptosis by Flow Cytometry

Before and after the loading of pathological cyclic strain, the PDL cells were gently treated with trypsin, washed once with serum-containing medium, and collected (5 × 10^5^) by centrifugation. The cells were suspended in 500 µL of 1× binding buffer, and 5 µL of annexin V-FITC and propidium iodide (PI) were added according to the manufacturer’s instructions (Biovision, Inc., Mountain View, CA, USA). After incubation at room temperature for 5 min in the dark, the cells were evaluated for annexin V-FITC and PI binding by flow cytometry using a FACSCalibur (BD Biosciences, Inc., San Jose, CA, USA).

### Western Blot Analysis

Cells cultured with or without cyclic strain on six-well plates were scraped into 300 µL of ice-cold lysis buffer (50 mM Tris–HCl, pH 7.4, 150 mM NaCl, 1 mM EDTA, 1% NP-40, 0.5% sodium deoxycholate, and 20 µL/mL protease inhibitor cocktail; Pharmingen, BD Biosciences). The samples were clarified by centrifugation at 13,000 rpm for 5 min at 4°C and boiled for 5 min with Laemmli sample buffer containing 100 mM NaF. Protein concentrations were determined by the Bradford method (Bio-Rad Laboratories, Richmond, CA, USA). Equivalent protein amounts were separated in 10% SDS–polyacrylamide gels and transferred to Immobilon-P polyvinylidene fluoride membranes (Millipore Corp., Bedford, MA, USA). The blots were then hybridized with specific primary antibodies and secondary antibodies labeled with an IRDye800-conjugated affinity-purified anti-rabbit/goat immunoglobulin M antibody (Rockland, Gilbertsville, PA, USA). The membrane was washed several times and scanned using an Odyssey infrared imaging system (LICOR, Lincoln, NE, USA) at a wavelength of 800 µm. The data were analysed with Odyssey software.

### RNA Interference and Inhibitor Treatment

The mRNA sequence of human RhoGDIα was acquired from NCBI GenBank. Small interfering RNAs (siRNAs) against human RhoGDIα were designed and synthesised by GenePharma Biological Company (Shanghai, China). The siRNA sequences targeting RhoGDIα were (5′→3′) GAGAUAGUGUCCGGCAUGAdTdT and UCAUGCCGGACACUAUCUCdTdT′. After incubation for 24 h, the cells were transfected with siRNA using Lipofectamine™ 2000 (Invitrogen, Life Technologies) at a final RNA concentration of 100 nM, according to the manufacturer’s instructions. After a 6-h incubation at 37°C in a humidified CO_2_ incubator, the transfection medium was replaced with DMEM for 18 h prior to induction of cyclic strain. Non-silencing siRNA was used as a negative control (N.C.).

For inhibitor studies, human PDL cells were pre-incubated with 10 µM z-DEVD-FMK, a specific caspase-3 inhibitor, for 1 h before cyclic strain was applied. PDL cells under the same conditions except without inhibitor were used as controls.

### Statistical Analysis

Data are presented as means ± standard deviation (SD) of three separate experiments. One-way ANOVA with the Student–Newman–Keuls test was used to compare values and to assess statistical significance (*p*≤0.05).

## Results

### Stretching Force Altered the Morphology of Human PDL Cells

**Figure 1:**
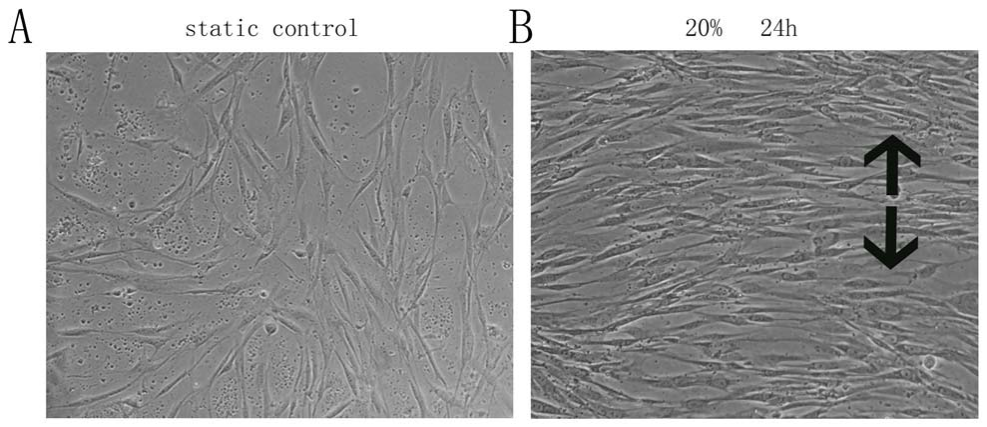
Pathological cyclic strain-induced morphological changes in human PDL cells. A. Cells cultured in the absence of cyclic strain are aligned multi-directionally. B. Cells subjected to 20% strain for 24 h are aligned primarily in one direction, with the long axis perpendicular to the cyclic strain force vector. The black arrow shows the direction of the stretching force (original magnification, ×200).

The *in vitro* application of cyclic stretching force altered the morphology of PDL cells. The cells became parallel to one another and were aligned with the long axes perpendicular to the stretching force vector (Fig. 1).

### Analysis of Apoptosis in Human PDL Cells by Annexin V and PI Staining

**Figure 2:**
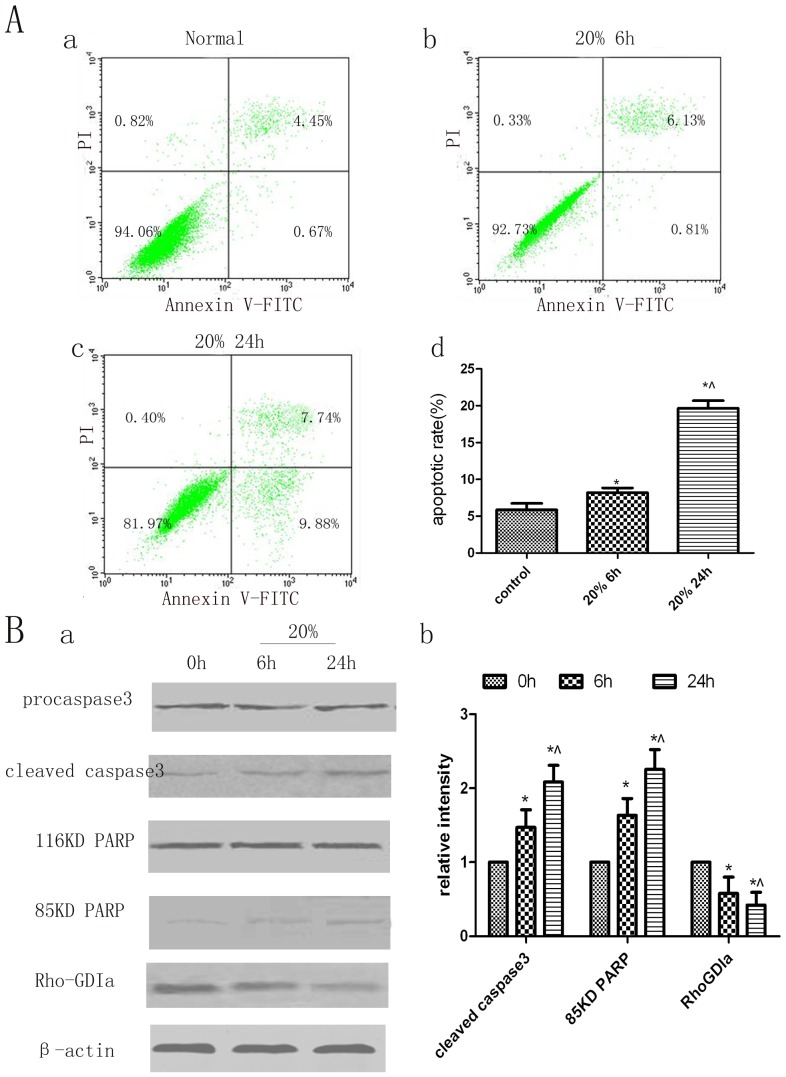
Analysis of apoptosis and protein changes in human PDL cells under pathological cyclic strain. A. Flow cytometric analysis of apoptosis in human PDL cells stained with annexin V and PI. Dot plots of cells stained with annexin V and PI are shown. The lower right quadrant indicates cells in early stages of apoptosis, and the upper right quadrant indicates cells in late stages of apoptosis. (a) Non-stretched group at 6 h. (b) 20% strain group at 6 h. (c) 20% cyclic strain group at 24 h. (d) Quantitation of apoptotic cells in each group. Bars represent means ± SD of at least three experiments. *p<0.05, static control group vs. 20% cyclic strain group at 6 h and 24 h; ^∧^ *p*<0.05, 20% cyclic strain group at 6 h vs. 24 h. B. Western blot analysis of RhoGDIα, caspase-3, and PARP protein levels. (a) Exposure to cyclic stretch correlates with increased levels of cleaved 19-kDa caspase-3 and 85-kDa PARP; the levels of 32-kDa procaspase-3 and 116-kDa PARP proteins remained unchanged, and the RhoGDIα level decreased. (b) Quantitative analysis of the protein levels of RhoGDIα, 85-kDa PARP, and cleaved caspase-3 in cell lysates of human PDL cells exposed to cyclic stretch for 6 and 12 h. Bars represent means ± SD of at least three experiments. *p<0.05, static control group vs. 20% cyclic strain group at 6 and 24 h; ^∧^ *p*<0.05, 20% cyclic strain group at 6 h vs. 24 h.

Apoptotic cells were identified by double labelling with annexin V and PI or by labelling with only annexin V. PI labels all dead cells, including necrotic cells and cells in late stages of apoptosis; cells entering early apoptosis are stained only by annexin V, and viable cells do not stain with annexin V or PI. Figure 2A shows representative dot plots of annexin V and PI staining. The lower left quadrant indicates viable cells (V-FITC−/PI−); the lower right quadrant, cells in early apoptosis (V-FITC+/PI−); the upper right quadrant, cells in late apoptosis (V-FITC+/PI+); and the upper left quadrant, necrotic cells (FITC−/PI+). In human PDL cells subjected to 20% cyclic strain for 6 h, the rate of apoptosis (cells in early and late stages of apoptosis) was increased slightly, by about 2%, compared with that in control cells. After 24 h of cyclic strain, the apoptosis rate was increased significantly, by about 14%, mainly due to cells entering early apoptosis.

### Cleavage of Caspase-3 and PARP Increased and RhoGDIα Decreased after Application of Cyclic Stretching Force

As RhoGDIα may be involved in apoptosis of human PDL cells subjected to 20% cyclic strain, the effect of cyclic strain on RhoGDIα expression was examined. Compared with static control cells, PDL cells subjected to 20% strain at the frequency of 0.1 Hz for 6 or 24 h, respectively, showed lower levels of RhoGDIα protein expression on Western blots (Fig. 2B). In contrast, the expression of cleaved caspase-3 and PARP was increased under cyclic strain compared with the expression in static control cells (Fig. 2B).

### Knock-down of RhoGDIα Sensitises Human PDL Cells to Cyclic Strain-induced Apoptosis and Increases Apoptosis in a Caspase-dependent Manner

**Figure 3:**
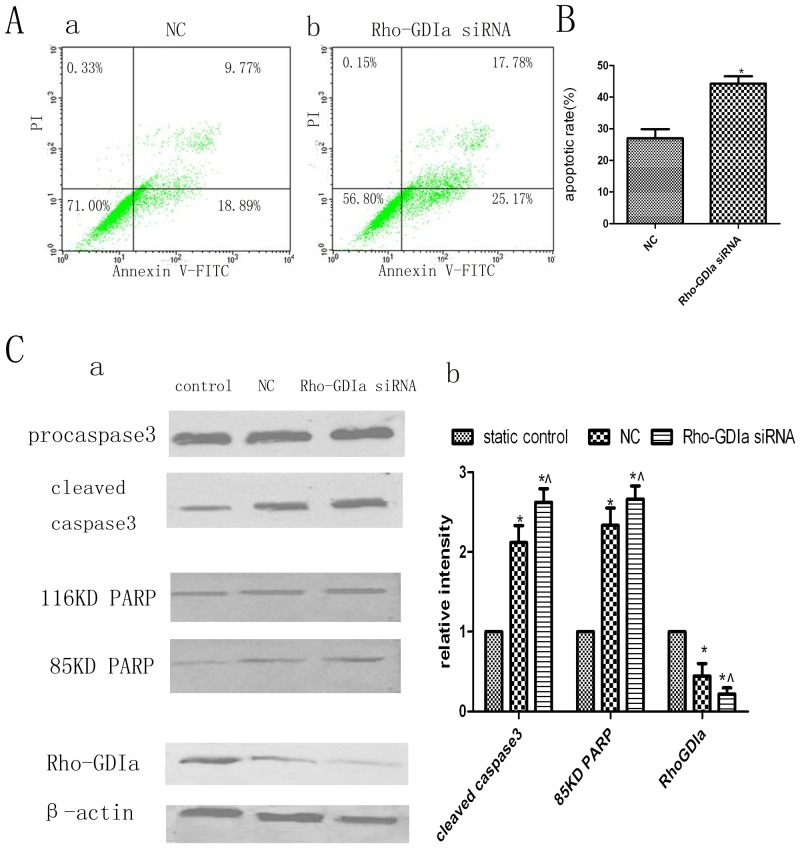
Analysis of apoptosis and protein changes after knock-down of RhoGDIα. A. Apoptosis of human PDL cells subjected to 20% cyclic strain for 24 h was determined by flow cytometry of cells stained with annexin V and PI. Cells transfected with non-silencing siRNA (which did not recognise any known human gene) were used as a negative control (N.C. group). (a) N.C. group. (b) RhoGDIα knock-down group. B. Quantitation of apoptotic cells in each group. The apoptosis rate of RhoGDIα knock-down cells was 1.5 to 2 times the rate of N.C. cells. Bars represent means ± SD of at least three experiments. *p<0.05, N.C. group vs. RhoGDIα-siRNA group. C. Western blot analysis of RhoGDIα, caspase-3, and PARP protein levels. (a) Compared with N.C. cells, RhoGDIα knock-down cells exhibited increased cleavage of caspase-3 and PARP; the levels of 32-kDa procaspase-3 and 116-kDa PARP proteins were unchanged, and the level of RhoGDIα was decreased. (b) Quantitative analysis of the levels of RhoGDIα, 85-kDa PARP, and cleaved caspase-3 in each group. Bars represent means ± SD of at least three experiments. *p<0.05, static control group vs. N.C. and RhoGDIα-siRNA groups. ^∧^ *p*<0.05, N.C. group vs. RhoGDIα-siRNA group.

Next, we examined the effect of RhoGDIα knock-down on apoptosis. Cells with RhoGDIα knock-down exhibited an apoptosis rate of approximately 43% before the application of cyclic strain, as measured by flow cytometry, whereas the apoptosis rate of the negative control cells was approximately 28% (Fig. 3A). In addition, compared with the negative control cells, the RhoGDIα knock-down cells exhibited increased cleavage of caspase-3 and PARP by Western blot analysis (Fig. 3B).

### Inhibition of Caspase-3 Confers Resistance to Apoptosis in Human PDL Cells, but does not Decrease Apoptosis Via Other Signalling Pathways

**Figure 4:**
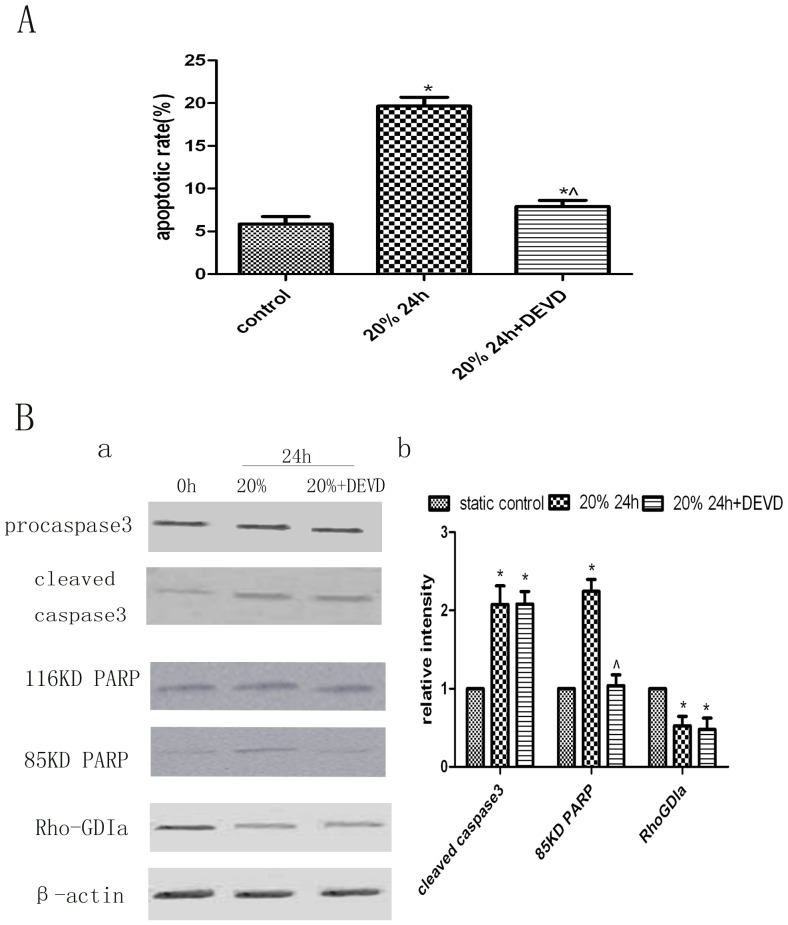
Analysis of apoptosis and protein changes after treatment with a specific caspase-3 inhibitor. A. Quantitation of apoptotic cells in each group. The number of apoptotic cells in the group subjected to 20% cyclic strain increased with time. Treatment with 10 µM z-DEVD-FMK, a caspase-3-specific inhibitor, conferred resistance to apoptosis in human PDL cells, but did not affect apoptosis via other signalling pathways. Bars represent means ± SD of at least three experiments. *p<0.05, static control group vs. 20% cyclic strain group at 24 h and 20% +DEVD group at 24 h groups; ^∧^ *p*<0.05, 20% cyclic strain group at 24 h vs. 20% +DEVD group at 24 h. B. Western blot analysis of RhoGDIα, caspase-3, and PARP protein levels. (a) Cells subjected to 20% cyclic strain in the presence of z-DEVD-FMK exhibited decreased cleavage of PARP compared with the level in the 20% cyclic strain group in the absence of z-DEVD-FMK, while the protein levels of RhoGDIα and 19-kDa caspase-3 did not differ significantly. In addition, the 20% +DEVD group exhibited almost no difference in PARP cleavage compared with the static control group, but showed higher RhoGDIα and cleaved caspase-3 protein levels. (b) Quantitative analysis of the protein levels of RhoGDIα, 85-kDa PARP, and cleaved caspase-3 in each group. Bars represent means ± SD of at least three experiments. *p<0.05, static control group vs. 20% cyclic strain group at 24 h and 20% +DEVD group at 24 h groups; ^∧^ *p*<0.05, 20% cyclic strain group at 24 h vs. 20% +DEVD group at 24 h.

Some human PDL cells were incubated with z-DEVD-FMK for 1 h to inhibit caspase-3 before applying cyclic strain. The cells that underwent 20% cyclic strain after incubation with z-DEVD-FMK (20% +DEVD group) exhibited about 12% less apoptosis than cells that underwent 20% cyclic strain in the absence of z-DEVD-FMK (20% −DEVD group) and about 2% more apoptosis than the static control group (Fig. 4A). The protein levels of RhoGDIα and cleaved caspase-3 did not differ significantly between the 20% +DEVD and 20% −DEVD groups, but the 20% +DEVD group exhibited decreased cleavage of PARP. Compared with the static control group, the 20% +DEVD group showed almost no difference in PARP cleavage, but had higher levels of RhoGDIα and cleaved caspase-3 (Fig. 4B).

## Discussion

Many kinds of cells in the periodontium, including PDL cells (e.g., fibroblasts, cementoblasts, and osteoblasts), vascular cells, and hematopoietic cells, participate in periodontal remodelling. Human PDL cells play important roles in not only maintenance of the periodontium but also promotion of periodontal regeneration [24], [25].

The ability of human PDL cells to sense and respond to physical stresses such as occlusal force is required for periodontal tissue homeostasis and normal development. In the periodontium, applied forces of physiological magnitude regulate cellular processes that are critical for normal tissue maintenance [26]. In contrast, forces of pathological magnitude can induce apoptosis [21], [27]. In this study, we used a Flexcell Tension Plus system, which can produce different stretching strains, to exert a cyclic stretching force on cells that was probably analogous to the stresses on PDL fibroblasts *in vivo*. We hypothesised that our experimental design would enable us to clarify how stretching force induces apoptosis.

To quantify human PDL cell apoptosis induced by pathological cyclic strain, we used flow cytometry to count apoptotic cells stained with annexin V and PI. We found that the extent of apoptosis caused by pathology cyclic strain is time-dependent. The extent of apoptosis caused by pathological cyclic strain was time-dependent, with a higher rate of early apoptotic cells at 24 h than at 6 h of pathological cyclic strain. According to this result and clinical application, human PDL cells should not be subjected to a constant pathological level of cyclic strain (excessive orthodontic force) for longer than 24 h, or the cells may irreversibly enter late apoptosis or even undergo necrosis.

The mechanism by which human PDL cells convert cyclic strain to biochemical signals has not yet been elucidated. The previous proteomic analysis on low shear stress-induced vascular remodelling demonstrated that RhoGDIα can respond to shear stress and modulate vascular smooth muscle cell migration and apoptosis [20]. Hence, it was hypothesised that RhoGDIα may participate in the recognition and transduction of extracellular cyclic strain stimuli in human PDL cells. RhoGDIα is a member of the Rho GDP dissociation inhibitors, which have been shown to negatively regulate the activities of small G proteins of the Rho family by inhibiting GDP (inactive)/GTP (active) cycling and cytosol (inactive)/membrane(active) translocation [28], [29]. Our findings demonstrate that cyclic strain downregulated the protein level of RhoGDIα. The knock-down of RhoGDIα by targeted siRNA was accompanied by enhanced PDL cell apoptosis and increased levels of cleaved caspase-3 and PARP. These results suggest that RhoGDIα has a significant role in the mechanotransduction and functional regulation of human PDL cells in response to cyclic strain, which is consistent with its function in cancer cells and other normal cells [19], [20]. This suggests that higher expression of RhoGDIα may be beneficial for preventing apoptosis of human PDL cells.

A recently published report revealed that cyclic stretch-induced apoptosis may be controlled by caspase-3, considering its importance in apoptosis [22]. In the current study, pathological cyclic strain induced a time-dependent increase in cleaved caspase-3 protein levels, and this was associated with increased apoptosis of cultured human PDL cells. A caspase-3-specific inhibitor, z-DEVD-FMK, significantly decreased the level of cleaved PARP and downregulated apoptosis. These data lead us to conclude that z-DEVD-FMK could inhibit only apoptosis mediated through caspase-3 signalling pathways and not apoptosis mediated by other signalling pathways.

Caspase-3 and its substrate, PARP, are key modulators of apoptosis, especially through the generation of the 85-kDa product generated by cleavage of PARP by caspase-3. To confirm our flow cytometry findings, we analysed the protein levels of PARP and caspase-3 on Western blots. The levels of 85-kDa cleaved PARP and 19-kDa cleaved caspase-3 increased in a time-dependent manner in stretched cells. In the presence of z-DEVD-FMK, The level of 19-kDa cleaved caspase-3 in stretched cells was the same in the absence and presence of z-DEVD-FMK and was significantly higher than the level in the non-stretched control group. As z-DEVD-FMK does not inhibit the cleavage of procaspase-3 to caspase-3, we concluded that its inhibitory effects on cyclic stretch-induced apoptosis of human PDL cells occur primarily at the post-cleavage level [30].

Human PDL cells subjected to stretching also exhibited morphological changes. The cells were longer than non-stretched control cells, but were still spindle-shaped, and became aligned perpendicular to the stretching force vector, as has been reported previously [31], [32]. This orientation of human PDL cells in response to cyclic stretching may represent a self-protection mechanism as it would prevent the cells from becoming excessively elongated and thus reduce the probability of injury. When mechanical force is applied to teeth, the alignment of PDL cells perpendicular to the strain force vector may be essential for maintaining PDL architecture [33], [34].

**Figure 5:**
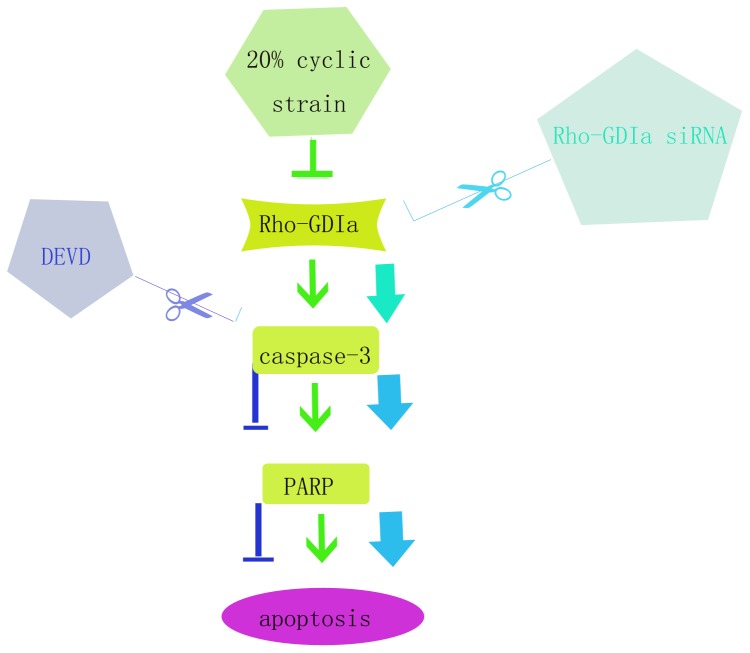
Schematic of the RhoGDIα/caspase-3/PARP pathway for the mediation of enhanced apoptosis induced by pathological cyclic strain. Inhibition of the proteins in the RhoGDIα/caspase-3/PARP pathway regulates the effect of cyclic strain on downstream, but not upstream, members of the pathway. The present study elucidates the hierarchical relationships among the signalling molecules shown in this diagram.

In conclusion, we provide evidence for a novel function of RhoGDIα in the protection of human PDL cells against apoptosis induced by cyclic strain. We demonstrated that RhoGDIα activity is a potent inhibitor of PARP cleavage by caspase-3 both *in vitro* and during apoptosis (Fig. 5). The present findings offer novel insight into the mechanism of apoptosis induced by pathological cyclic strain in human PDL cells through the activation of caspase-3 via a RhoGDIα signalling cascade. Our future studies will focus on identifying related anti-apoptotic molecules that have significance for understanding mechanical stress in oral medicine.

## Supporting Information

Figure S1 **Analysis of apoptosis in human PDL cells under 20% cyclic strain for 6 h and 24 h.** 1: presents the first donor. 2: presents the second donor. 3: presents the third donor.
(TIF)

Figure S2 **Analysis of apoptosis in human PDL cells after knock-down of RhoGDIα.** 1: presents the first donor. 2: presents the second donor. 3: presents the third donor.
(TIF)

Figure S3 **Analysis of apoptosis in human PDL cells after treatment with a specific caspase-3 inhibitor.**1: presents the first donor. 2: presents the second donor. 3: presents the third donor.
(TIF)

Figure S4 **Analysis of protein changes in human PDL cells under 20% cyclic strain for 6 h and 24 h.**1: presents the first donor. 2: presents the second donor. 3: presents the third donor.
(TIF)

Figure S5 **Analysis of protein changes in human PDL cells after knock-down of RhoGDIα.** 1: presents the first donor. 2: presents the second donor. 3: presents the third donor.
(TIF)

Figure S6 **Analysis of protein changes in human PDL cells after treatment with a specific caspase-3 inhibitor.**1: presents the first donor. 2: presents the second donor. 3: presents the third donor.
(TIF)
