# Supplementary figures and images for: Pathological Cyclic Strain-Induced Apoptosis in Human Periodontal Ligament Cells through the RhoGDIα/Caspase-3/PARP Pathway
Source: PLoS One. 2013 Oct 10;8(10):e75973. doi: 10.1371/journal.pone.0075973 (PMC3794943; doi:10.1371/journal.pone.0075973)

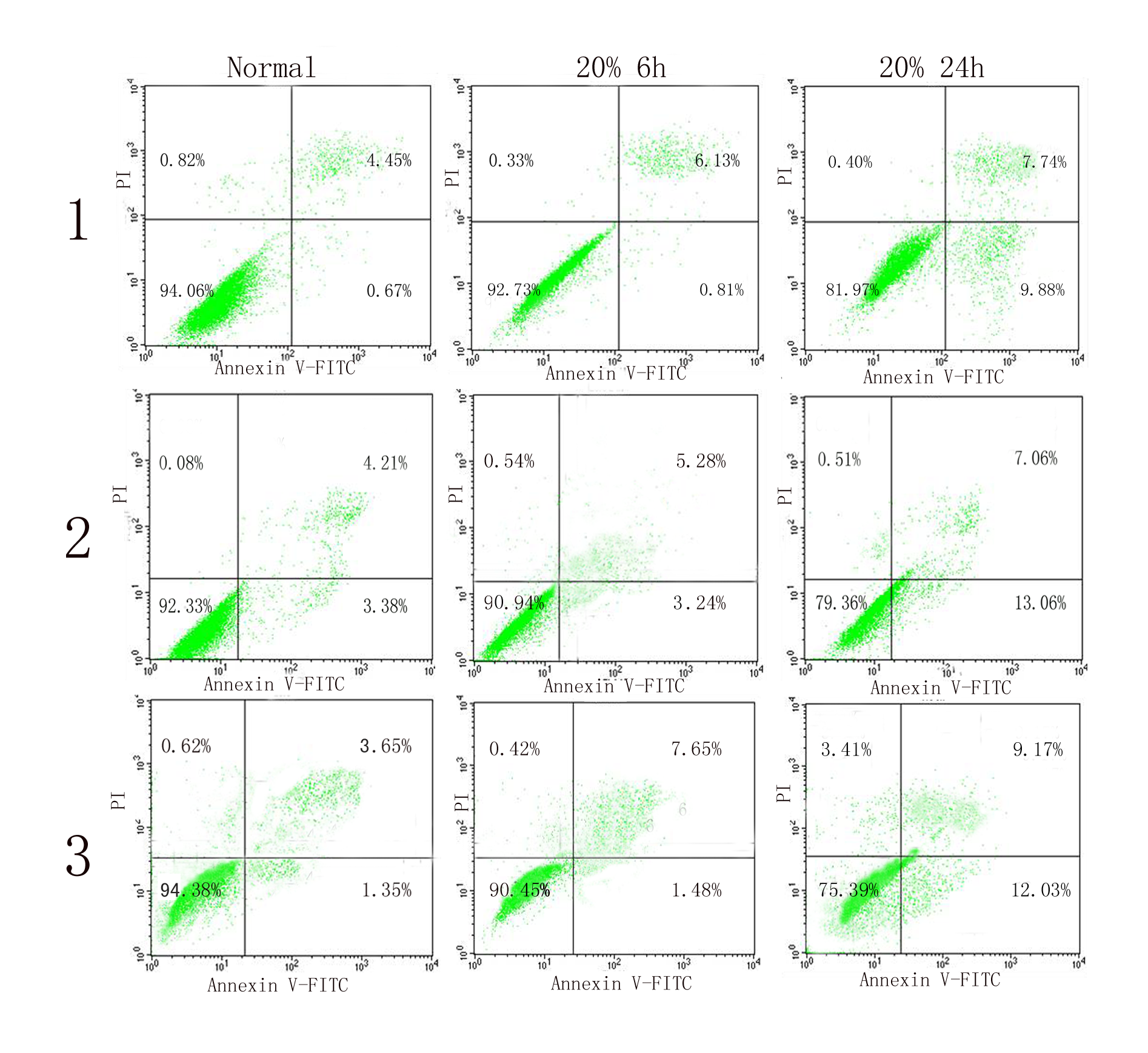

Supplement: Figure S1 — Analysis of apoptosis in human PDL cells under 20% cyclic strain for 6 h and 24 h. 1: presents the first donor. 2: presents the second donor. 3: presents the third donor. (TIF) [file pone.0075973.s001.tif]

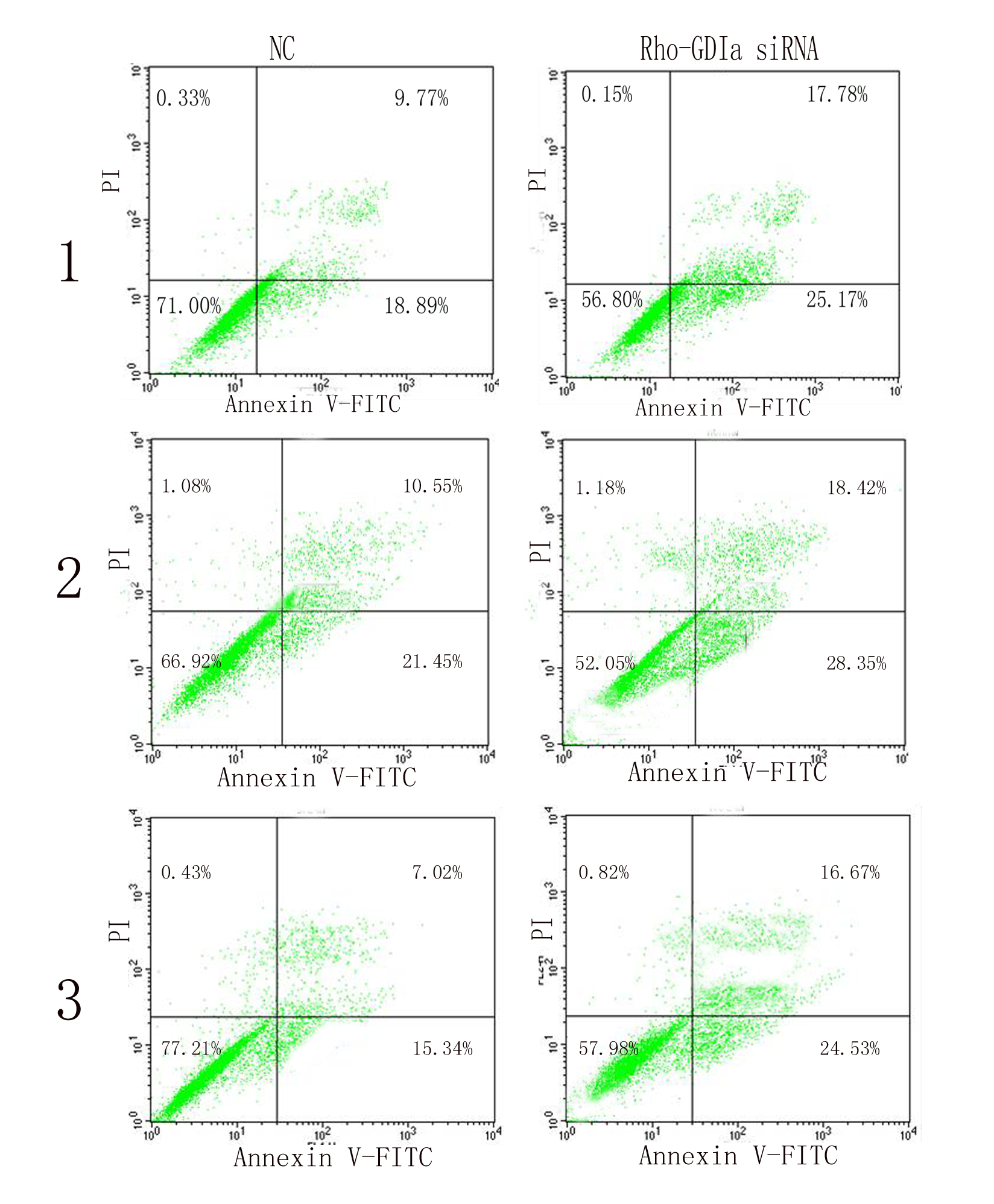

Supplement: Figure S2 — Analysis of apoptosis in human PDL cells after knock-down of RhoGDIα. 1: presents the first donor. 2: presents the second donor. 3: presents the third donor. (TIF) [file pone.0075973.s002.tif]

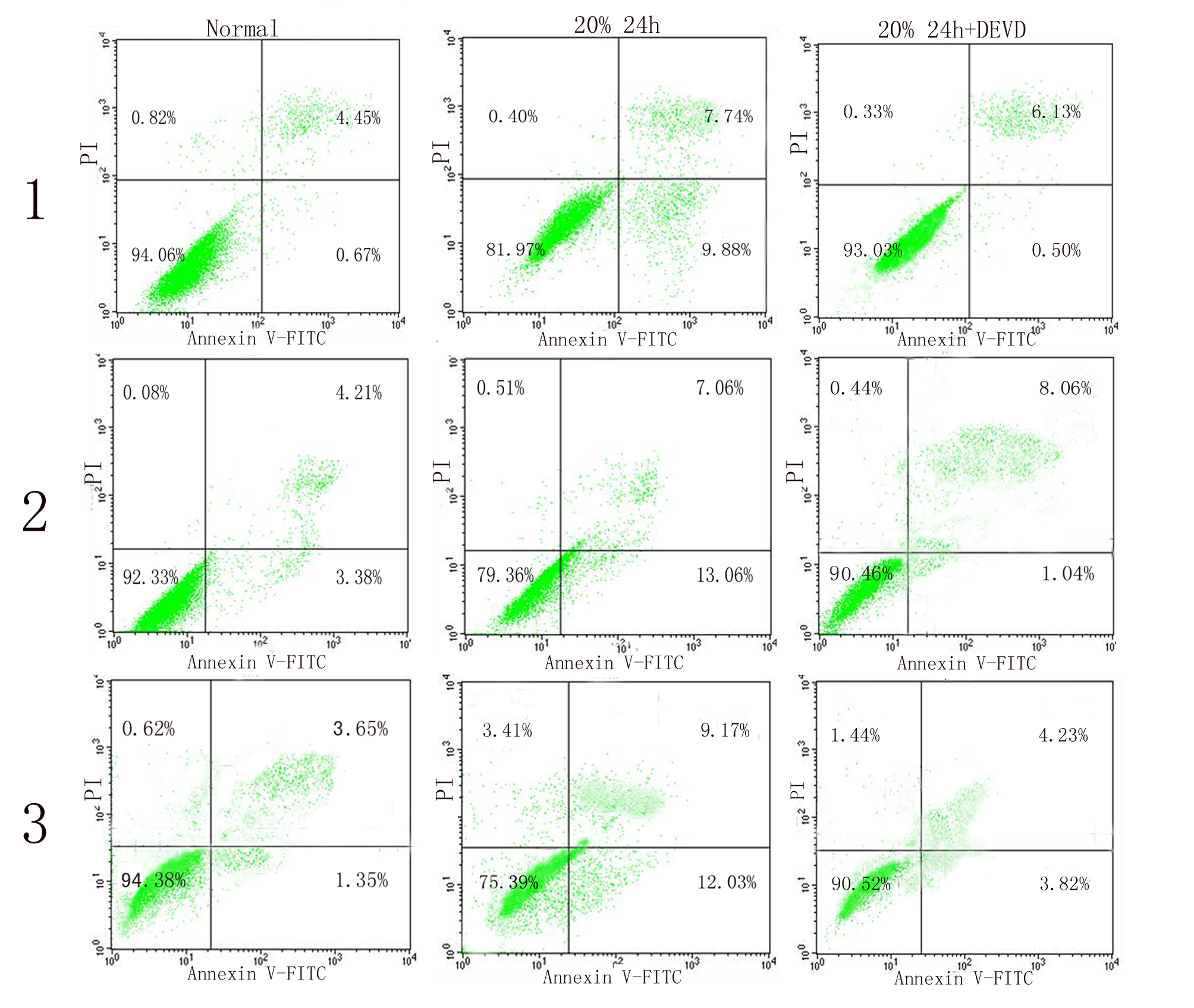

Supplement: Figure S3 — Analysis of apoptosis in human PDL cells after treatment with a specific caspase-3 inhibitor.1: presents the first donor. 2: presents the second donor. 3: presents the third donor. (TIF) [file pone.0075973.s003.tif]

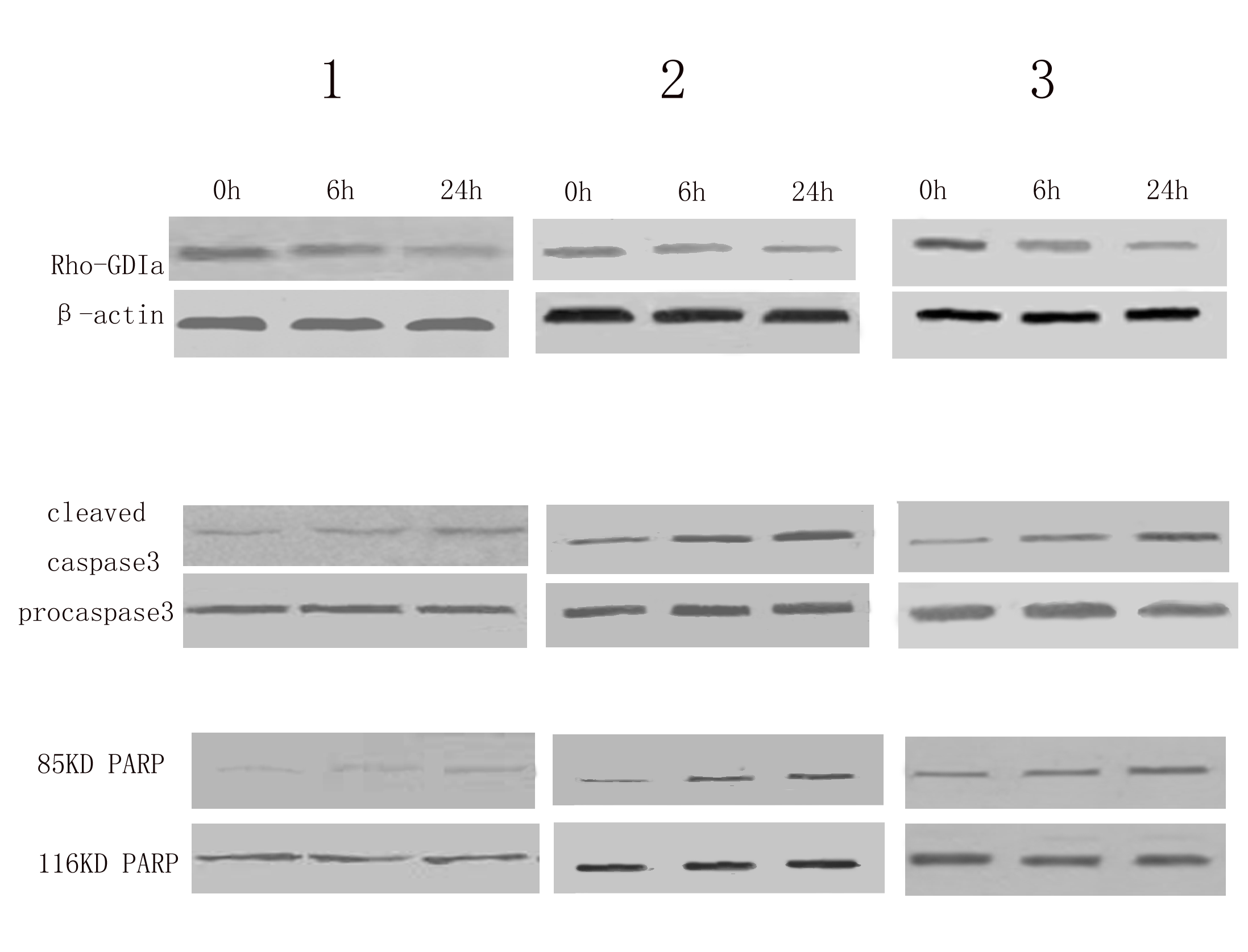

Supplement: Figure S4 — Analysis of protein changes in human PDL cells under 20% cyclic strain for 6 h and 24 h.1: presents the first donor. 2: presents the second donor. 3: presents the third donor. (TIF) [file pone.0075973.s004.tif]

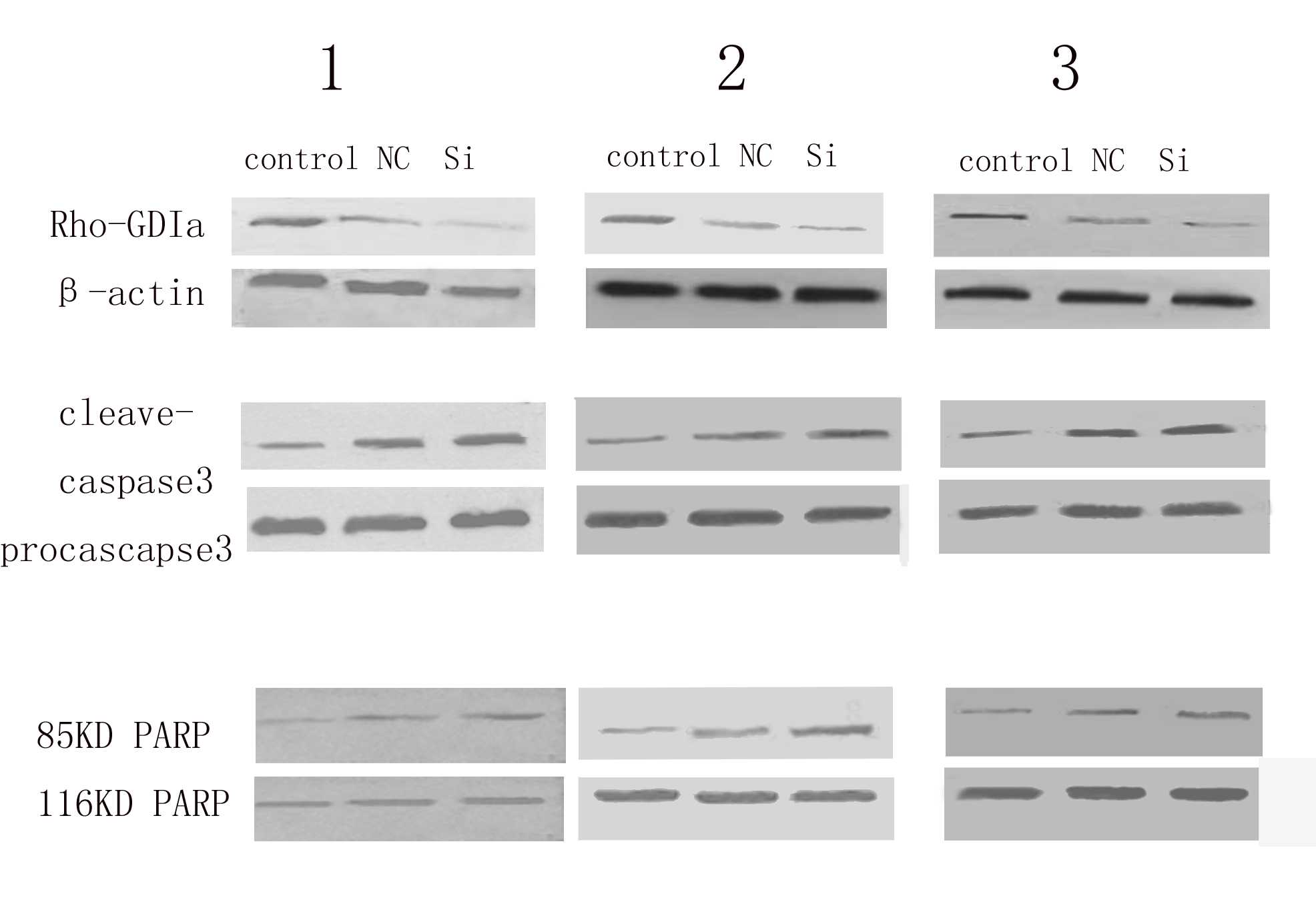

Supplement: Figure S5 — Analysis of protein changes in human PDL cells after knock-down of RhoGDIα. 1: presents the first donor. 2: presents the second donor. 3: presents the third donor. (TIF) [file pone.0075973.s005.tif]

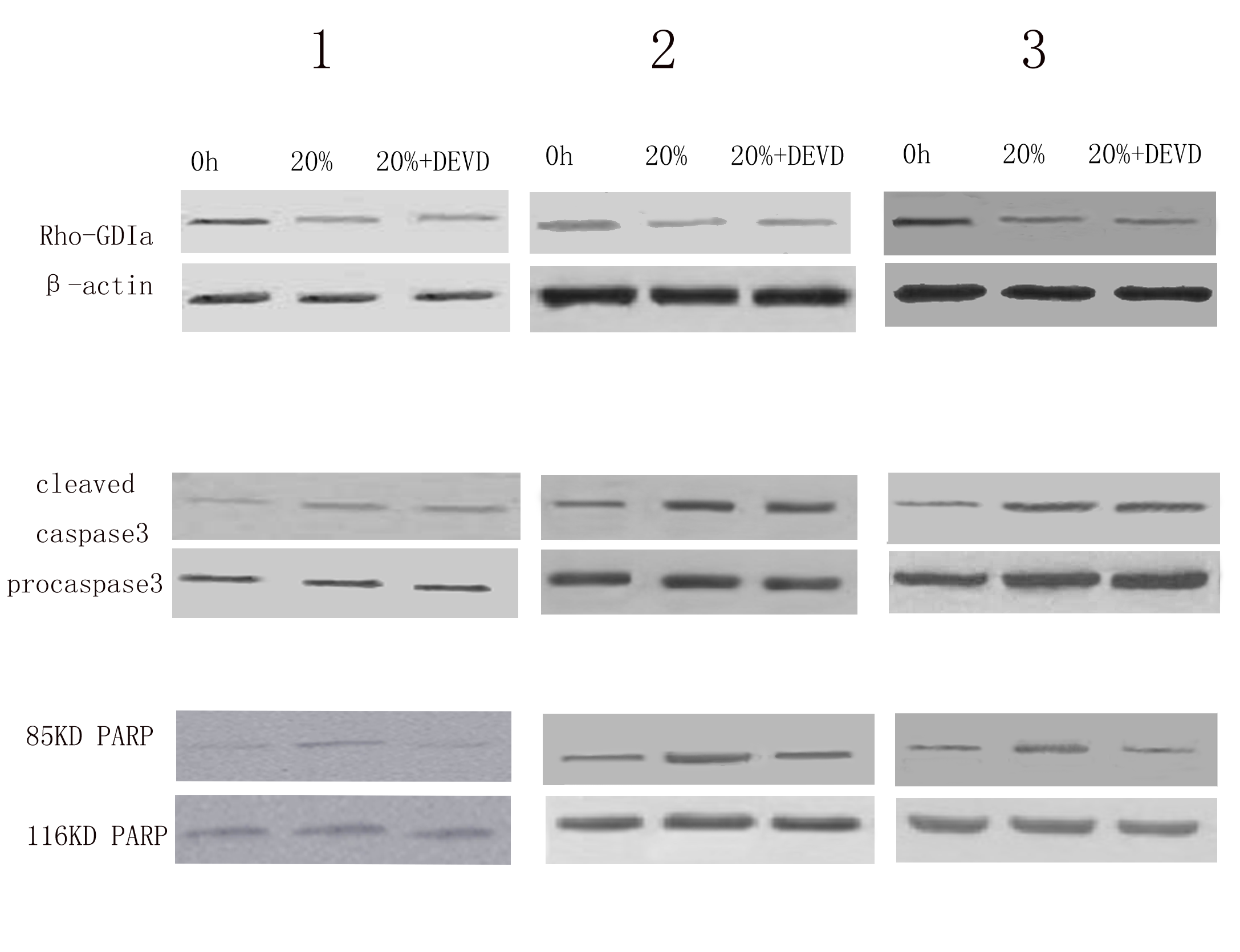

Supplement: Figure S6 — Analysis of protein changes in human PDL cells after treatment with a specific caspase-3 inhibitor.1: presents the first donor. 2: presents the second donor. 3: presents the third donor. (TIF) [file pone.0075973.s006.tif]
